# Evaluating a learning management system for blended learning in Greek higher education

**DOI:** 10.1186/s40064-016-1705-8

**Published:** 2016-02-01

**Authors:** Katerina Kabassi, Ioannis Dragonas, Alexandra Ntouzevits, Tzanetos Pomonis, Giorgos Papastathopoulos, Yiannis Vozaitis

**Affiliations:** TEI of Ionian Islands, M. Minotou-Giannopoulou, 29100 Zakynthos, Greece; Department of Informatics, TEI of Athens, Athens, Greece

**Keywords:** Blended learning, Learning management systems, e-Learning

## Abstract

This paper focuses on the usage of a learning management system in an educational institution for higher education in Greece. More specifically, the paper examines the literature on the use of different learning management systems for blended learning in higher education in Greek Universities and Technological Educational Institutions and reviews the advantages and disadvantages. Moreover, the paper describes the usage of the Open eClass platform in a Technological Educational Institution, TEI of Ionian Islands, and the effort to improve the educational material by organizing it and adding video-lectures. The platform has been evaluated by the students of the TEI of Ionian Islands based on six dimensions: namely student, teacher, course, technology, system design, and environmental dimension. The results of this evaluation revealed that Open eClass has been successfully used for blended learning in the TEI of Ionian Islands. Despite the instructors’ initial worries about students’ lack of participation in their courses if their educational material was made available online and especially in video lectures; blended learning did not reduce physical presence of the students in the classroom. Instead it was only used as a supplementary tool that helps students to study further, watch missed lectures, etc.

## Background

With the rapid increase in Internet usage, online instruction is now widely adopted in universities and other educational organizations (Huang et al. 2012). The online learning environments provide flexibility for students regarding time and place as well as a self-paced learning (Terrell and Dringus 2000; Graff 2003; Virvou and Alepis 2013; Alepis and Virvou 2014). As a result, many online activities have come to influence traditional learning. This new form of learning is called blended learning. Indeed, blended learning classes are the classes where face to face and online activities are integrated in a planned, pedagogically valuable manner and online activities replace some face to face time (So and Brush 2008; Olapiriyakul and Scher 2006; Picciano 2009).

The evolving symbiosis of technology with traditional pedagogical approaches, facilitating content richness, flexible content access and alternative communication channels, may benefit the learning process (Nikolaidou et al. 2010). Researchers have argued about the advantages of the combination of face to face teaching and online learning and emphasize on the promotion of learner-centered, active and constructive learning (O’Donnell et al. 2006; Salomon and Ben-Zvi 2006; Stahl 2006; Giannousi et al. 2014). As a result, the use of computer-mediated communication (CMC) and e-Learning tools increases in higher education (Kanuka et al. 2002; Rovai 2002; Stodel et al. 2006). According to a survey of the National Postsecondary Education Cooperative in 2006 and 2007, 61 % of degree-granting postsecondary institutions run online courses (Parsad et al. 2008).

For this purpose, different systems have been developed for e-Learning. The systems that seem to provide a better solution to higher educational institutions are the learning management systems (LMSs). The main characteristics of these systems are that they are not static, are easily reusable and, therefore, can successfully address the needs of a higher educational institution such as a university that offers a variety of courses. LMSs provide the instructors the capability of designing and administrating their courses as they want. In this way the blended learning is better supported. For these purposes, a LMS was selected for Technological Educational Institutions (TEIs) in Greece, which offers not only theoretical lessons but workshops as well.

In the last decade, in Greece, there was an effort to employ LMSs in all universities and TEIs. In this paper, we try to review the usage of LMS in different institutions for higher education in Greece and focus on the case of the TEI of Ionian Islands. The TEI of Ionian Islands is a higher education institution that has special character due to the fact that its departments spread across three different islands between Greece and Italy. Indeed, as Pituch and Lee (2006) point out, e-Learning systems that facilitate blended learning may better accommodate the needs of learners or instructors who are geographically dispersed and have conflicting schedules.

Taking into account other definitions of blended learning, that introduce it as a combination of multiple delivery media designed to complement each other to promote the learning activity (Singh 2003), we have enhanced the learning experience by incorporating video-lectures in the LMS. Video is a rich and powerful medium being used in e-Learning as it can present information in an attractive and consistent manner (Zhang et al. 2006).

As blended e-Learning systems emerge as perhaps the most prominent instructional delivery solution, it is vital to explore what determines learning satisfaction (Wu et al. 2010). Therefore, the LMS used in the case study described has been evaluated. In an e-Learning environment, several factors account for users’ satisfaction. Those factors can be categorized into six dimensions: student, teacher, course, technology, system design, and environmental dimension (Sun et al. 2008). In view of these dimensions, we have implemented an evaluation experiment of a LMS used in the TEI of Ionian Islands with the participation of students, who are the real users of the system.

The rest of the paper is organized as follows: “Blended learning and learning management systems” section discusses related work in blended learning and LMSs in general. In “LMS in higher education in Greece” and “Evaluating the use of LMS in higher education in Greece” sections, we try to analyze the usage of LMSs in higher education in Greece taking into account the publications in the past. In “The case of TEI of Ionian Islands” and “Evaluation experiment” sections, we describe the case of Ionian Islands and the evaluation experiment conducted with the participation of real users. Finally, in the last sections we discuss the conclusions drawn by this work.

## Blended learning and learning management systems

The integration of traditional classroom methods with online activities is called blended learning (Graham 2006; Macdonald 2008; López-Pérez et al. 2011). It has been suggested by many researchers that blended learning methods are effective in facilitating the process of online collaborative learning (Carr-Chellman et al. 2000; Gabriel 2004; So and Brush 2008). However, the success of blended learning is not only the result of the simple integration of two different forms of learning (De George-Walker and Keeffe 2010). The main advantages of this new form of learning mainly appear in situations where the number of students is high and, therefore, the existence of Information and Communication Technologies (ICTs) provides the opportunity to comprehend and extend the knowledge presented in a more efficient way (Singh 2010). For this purpose, blended learning models have been used more extensively in higher education than in lower levels of education (Garrison and Kanuka 2004).

Indeed, the use of different methods in teaching and learning enables students to comprehend the subject being taught better, clarify the rules and goals of the course and gives them a self-paced learning and greater flexibility (Ginns and Ellis 2007; Macedo-Rouet et al. 2009; López-Pérez et al. 2011). A closer examination reveals the ability of asynchronous Internet communication technology to facilitate a simultaneously independent and collaborative learning experience (Garrison and Kanuka 2004). However, there are also studies reporting that students may encounter difficulties in adjusting to blended learning (e.g. Bonk et al. 2002).

Blended learning is supported by learning management systems (LMSs), which are mainly Course Management Systems (CMSs) that are extensively used for supporting blended learning. Wilen-Daugenti (2008) interchanges the terms CMS and LMS. Gagné et al. (2005) define a CMS as a system that provides the tools for the development and delivery of courses, which are placed onto the Internet, whereas they define a LMS as a management system delivering online learning. Generally, LMSs are scalable systems, which can be used to support an entire university’s teaching and learning programs (Coates et al. 2005).

The main tools that all LMSs provide are:

- Asynchronous and synchronous communication
- Content development and delivery
- Formative and summative assessment

The asynchronous and synchronous communication may involve announcement areas, e-mail, chat, forums etc. The content development and delivery may involve learning resources, learning objects, files, links to internet resources, etc. Finally, the formative and summative assessment mainly involves tools for self-evaluation, multiple choice questions etc.

As a result, many LMSs have been developed for supporting blended learning such as WebCT (http://www.webct.com) or Cyber University of NSYSU (http://cu.nsysu.edu.tw). These systems can provide instructors and learners with multiple, flexible instructional methods, educational technologies, interaction mechanisms or learning resources, which they can apply in an interactive learning environment to overcome the limitations of classroom and e-Learning (Wu et al. 2010). In particular the LMSs that have been more extensively used in Greece are Moodle, Blackboard and Open eClass.

Blackboard (http://www.blackboard.com) provides traditional instruction and powers pure distance learning providing specific utilities (Yaskin and Everhart 2002) such as content management and sharing, assessment management, gradebook and assignment management, collaboration and communication, student and instructor portfolio, etc. Blackboard has been extensively used in educational institutions in North America. Indeed, in a study in 2003 that involved 113 educational institutions all over the world, Blackboard is identified as a major American-based LMS (Paulsen 2003).

Although Blackboard is an effective and efficient LMS, it also has some drawbacks. As Ng (2008) points out some resources published in Blackboard cannot be shared by other users due to copyright problems. Another technical limitation is associated with the management of hyperlinks for both internal and external documents.

Moodle, on the other hand, is an open-source learning platform designed to provide educators, administrators and learners with a single robust, secure and integrated system to create learning environments (http://www.moodle.org). The main advantage of Moodle in comparison to other commercial LMSs is that the courses developed are based on constructionist pedagogy (Veglis and Pomportsis 2005). Other advantages include:

- language packs in more than 80 languages,
- open source and therefore, free download, modification and distribution,
- different database software is supported,
- e-Learning standards such as SCORM are adopted,
- many Moodle’s themes allow Moodle to be used easily on mobile devices.

**Fig. 1:**
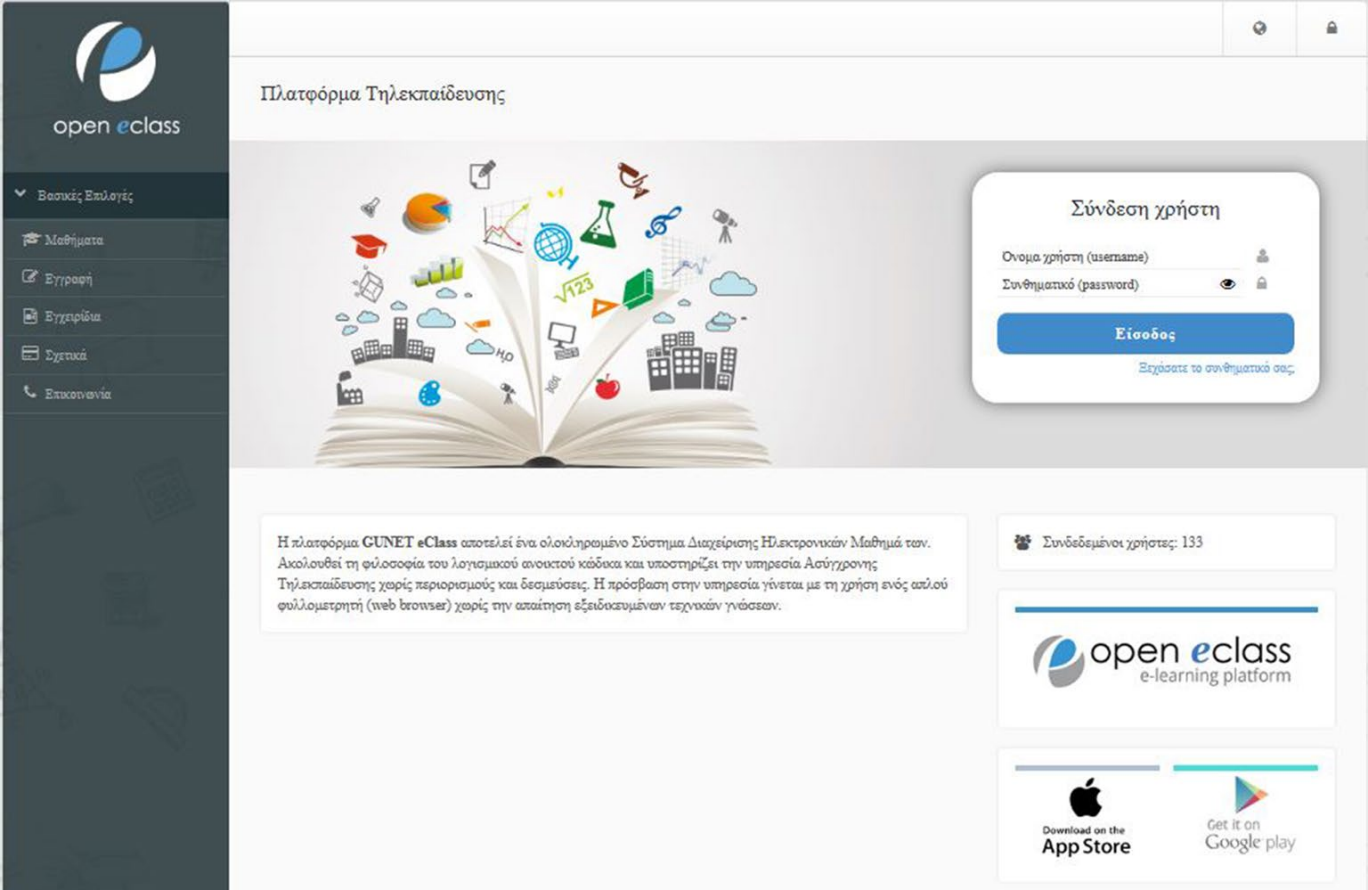
Screenshot of Open eClass

Finally, the Greek University Network (http://www.gunet.gr) has developed the platform Open eClass (http://eclass.gunet.gr) that is based on Claroline (http://www.claroline.net). This LMS is also open source and, therefore, free to download, modify and distribute. The platform has been translated in more than 35 languages and used in over 100 countries. The users can create learning paths compatible with the e-Learning standard SCORM and take advantage of a set of utilities for composing exercises, structuring agenda with tasks and deadlines, posting notifications, writing collaborative documents, performing asynchronous communication such as forums, linking to internet resources, publishing documents in any format, managing group, designing learning objects etc. (Fig. 1).

## LMS in higher education in Greece

The rapid uptake of campus-wide LMSs is changing the character of the on-campus learning experience (Coates et al. 2005). Indeed, according to several studies (e.g. Wang 2010) delivering information via the web is gaining popularity among both students and staff. LMS can support an entire university’s teaching and learning programs.

In Greece LMSs started to be used after 2000. For example, the Aristotle University of Thessaloniki (AUTh) installed Blackboard in 2003 (Veglis and Pomportsis 2005). In an effort to expand the usage of LMSs in higher education in Greece in a uniform way, the Greek University Network (GUNet) distributed the platform Open eClass. Furthermore, it provided support for the implementation of Open eClass in any institution by facilitating its installation and operation. In addition, Open eClass provides an internal structure for each lesson, which promotes communication between learners and educators, learning with active participation and ensuring open and free access to educational material (Papachristos et al. 2010).

As a result, Open eClass was adopted by most of the Universities and TEIs. Indeed, reports have been found for the usage of Open eClass in the TEI of Chalkida (Papazoglou et al. 2005; Spathopoulos 2007), Alexander TEI of Thesaloniki (Tzitzolaki et al. 2014), University of Thrace (Vernadakis et al. 2009), TEI of Epirus (Giannelou et al. 2005), TEI of Athens (Georgouli et al. 2005; Karolidis et al. 2005; Tsiakas et al. 2005), TEI of Lamia (Tziallas et al. 2005), Hellenic Open University (Papadakis et al. 2005), TEI of Crete (Vassilakis et al. 2005; Kalogiannakis et al. 2005), TEI of Larissa (Blanas 2008), Harokopio University (Chalkias and Anagnostopoulos 2004) etc.

Pange and Lekka (2012), in a pilot study examining the educational packages offered via Internet by Universities and TEIs in Greece, found out that 72 % of the randomly selected courses were delivered by Open eClass. Although the official LMS of most Universities and TEIs is Open eClass, the flexibility of Moodle as well as the fact that it is free to download, have made it attractive to many universities that use it as a secondary LMS. However, the use of Moodle is not official in most cases.

## Evaluating the use of LMS in higher education in Greece

Several studies (Ansorge and Bendus 2004; Boggs et al. 2004; Jones and Jones 2005; Vernadakis et al. 2009) reported that CMS have contributed positively to both instructional and learning needs. Therefore, in this section we will present different studies that are associated with the usage of Open eClass in Universities and TEIs in Greece.

More specifically, Kabouridis (2010) describes a study on the use of Open eClass in the department of Mechanical Engineering in the TEI of Patras. The study involved tutors that participated in a semi-structured interview and students that answered a questionnaire. The main conclusion of this study was that the stakeholders of the University in the twenty first century must realize that they cannot ignore the new academic framework of the e-Learning.

Similarly, Leventidis et al. (2005) and Georgouli et al. (2005) argue that the incorporation of e-Learning tools and, specifically, the open source e-Learning platform Open eClass has paved a new road in the educational procedure and has enriched the traditional teaching methods by encouraging the interaction among teachers, students and educational material.

The usage of Open eClass in the TEI of Chalkida is described in Papazoglou et al. (2005) and Spathopoulos (2007). More specifically, Spathopoulos (2007) conducted a study about the use of Open eClass in the Department of Aircraft Technology at the Technological Education Institute (TEI) of Chalkida and concluded that Open eClass was user-friendly and had easily and inexpensively been used to supplement a traditional classroom with a virtual one. This supplement had considerably reduced the amount of administration and management time required for the subjects taught and the students had been very positive as they felt that the teaching quality had been improved.

Chalkias and Anagnostopoulos (2004) and Nikolaidou et al. (2010) describe the usage of Open eClass in Harokopio University. The latter authors have conducted an evaluation study of the LMS with the participation of students, instructors and infrastructure-technology specialists to evaluate the ecosystem of blended learning. The most interesting observation of this research is that despite the instructors’ initial worries; blended learning did not reduce neither physical presence of the student in the classroom, nor face to face instructor–student communication. Vernadakis et al. (2009), on the other hand, in a study for the acceptance of the CMS in the University of Thrace, concluded that the usage of the CMS influenced negatively the physical participation of students in the courses.

Such worries were also noted in Tzitzolaki et al. (2014) who described a study on Open eClass with the participation of professors in the Alexander TEI of Thessaloniki. They observed that the subjects maybe did not use the ICT tools because they doubted if these tools reinforce the educational process or because of the intervention of some psychological factors (such as ignorance, fear or insecurity) affect their perceptions.

Positive attitude on the platform is reported in the study of Giannelou et al. (2005) in the TEI of Epirus. The study conducted by the Department of Informatics at TEI of Athens also reports very encouraging results about Open eClass usage (Georgouli et al. 2005) as students characterize Open eClass as user-friendly and this results in better adoptability. However, the main advantage of the e-Learning platform usage is that students were motivated to spend more time on their homework and they improved their scores. A different electronic study but concerning the same educational institution, is conducted by Tsiakas et al. (2005). The results of the study they conducted revealed that every innovation in the field of education attracts students’ interest and students must be encouraged to develop initiative and pursue knowledge, rather than merely react and absorb.

Similarly, students in the TEI of Crete appear to be more stimulated when they have to attend a course through Open eClass (Vassilakis et al. 2005; Kalogiannakis et al. 2005). In Vassilakis et al. (2005) study in the TEI of Crete, it is concluded that access to the distant resources is rapidly becoming common place and the platform’s users consider that they are more active and productive in the asynchronous e-Learning environment, although they have not fully exploited it yet.

Georgouli et al. (2006) attempted to analyze the use of the asynchronous e-Learning platform, Open eClass, at the TEI of Athens and compare the results of this analysis to observations made at the Universidade Nova de Lisboa that used Moodle. Open eClass has proven to be a valuable, extensible, versatile and powerful tool that can assist many educational tasks. Similar were the findings of the study of Tziallas et al. (2005) at TEI of Lamia, where it was reported that the simplicity of the platform and the fact that it is free are the main advantages for the usage of Open eClass.

Some studies focus more on the functionality of the platform. For example, Leventidis et al. (2005) analysed the various tools of the platform and found out that the most popular tools were agenda, announcements, assignments and forum. However, as Papachristos et al. (2010) in an extensive study on the use of the platform have observed, many features were not fully activated or updated. Additionally, in many cases the educational material was found to be either repetitive or very poor. Furthermore, the use of multimedia is rarely observed, as it is still at a basic level (e.g. presentation software, photographs, drawings, images) without often using animation or video.

Papachristos et al. (2010) also reported that another serious problem was the lack of availability of courses in foreign languages, which did not give any opportunity to attract international students. Other limitations were also found in a survey conducted by Lagiou et al. (2014) about the use of web 2.0 tools in adult education. Some of the problems identified involved semantic modelling of the course learning objectives and a mapping of how activities, materials and assignments address the learning objectives. They also noted the importance of mobile devices as a supplementary tool in their learning activities.

## The case of TEI of Ionian Islands

**Fig. 2:**
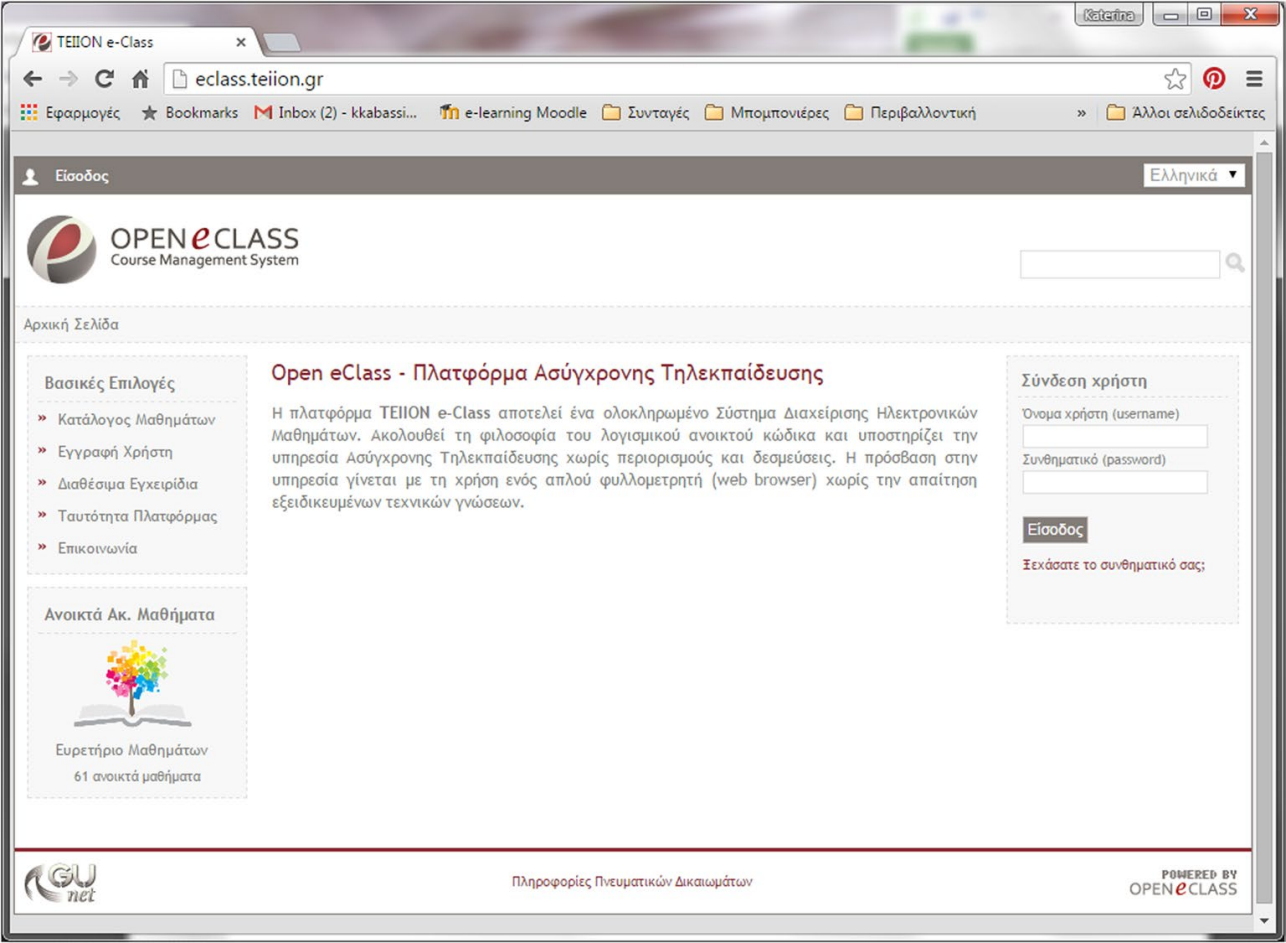
Screenshot of Open eClass in the TEI of Ionian Islands

TEI of Ionian Islands is a Higher Education Institution that has its departments spread across three different islands, namely Zakynthos, Cephalonia and Lefkada. These islands are located in the Ionian Sea, which is between Greece and Italy. The students are Greek from different parts of Greece. This special character of this educational institution increased the need for a LMS that provides the educational material through the internet to all students and instructors. Additionally, collaborative learning may be better supported by the use of such platforms (Fig. 2).

Open eClass was established in the TEI of Ionian Islands in 2006 and it has today 337 courses and 289 instructors that use the platform. Today, over 70 % of the institution’s available courses are supported through this platform. However, the instructors mainly use the platform as a supplementary tool for disseminating their files and they do not make use of all the platform’s features.

As a result, the last 2 years, the institution runs the open courses project (http://opencourses.teiion.gr/) that aims at introducing the platform to the tutors and the students and enhancing the teaching material provided in the courses provided by the LMS. For this purpose each department of the TEI of Ionian Islands uses Open eClass to provide blended learning to the students of these courses. There were courses that were organized to provide material mainly in text whereas for some other courses some video-lectures have been prepared. The tutors were selected to participate on the project based on their interest in having video-lectures as learning material. Furthermore, the project familiarized tutors with the copyright issues, either in disseminating their work or even in using third-party material.

The result of the project was that the LMS was significantly enhanced with updated material and provided video-lectures. Zhang et al. (2006) conducted an extensive research which revealed the benefits from e-Learning and especially video. Indeed, they report that video allows students to view actual objects and realistic scenes, to see sequences in motion, and to listen to narration.

Table 1 presents the departments of the educational institution and the courses being published in Open eClass at the end of winter and spring semester of academic year 2014–2015. At the last column of Table 1 one can see the instructors that have chosen to use Open eClass until the end of spring semester 2014–2015. One can easily notice that in all departments of TEI of Ionian Islands, there is an increase of the courses published in Open eClass. The departments that seem to use Open eClass more is Environmental Technology and Business Administration due to the fact that each of them incorporates two different faculties. Furthermore, the subject of the department seems to play an important role in the usage of the LMS. For example, the instructors of the department of Digital Media and Communication use the platform more extensively compared to the instructors of the department of Sound and Musical Instruments Technology or the department of Food Technology.

**Table 1:** Usage of Open eClass by the different departments in the TEI of Ionian Islands

Department	Courses in Open eClass winter semester 2014–2015	Courses in Open eClass spring semester 2014–2015	Instructors having published lessons in Open eClass until spring semester 2014–2015
Sound and Musical Instruments Technology	26	29	13
Business Administration	87	97	29
Food Technology	45	55	13
Environmental Technology	123	135	40
Digital Media and Communication	56	61	19

In terms of user activity, the platform visitors mainly target the two larger departments, namely Business Administration (39 %) and the department of Environmental Technology (34 %), which host most of the students, and also have the largest rates of LMS pervasion and acceptance. The departments of Food Technology and Digital Media and Communication also have adequate visitors (12 % each) and only the department of Sound Technology and Musical Instruments has limited visitors in its courses.

There is a significant increase of visitors during the 3 yearly exam periods, January–February, June and September, but also a constant number of visits during the semester’s period, which indicates exactly the complementarity and utilization of on-line courses in real-time teaching process.

## Evaluation experiment

In an e-Learning environment, several factors account for users’ satisfaction. Those factors can be categorized into six dimensions: student, teacher, course, technology, system design, and environmental dimension (Sun et al. 2008). Taking into account these dimensions a questionnaire was designed. The questions are based on those of Sun et al. (2008) but were designed to address the main issues in Greek Higher Education and provide some first conclusions despite the short time of the platform being updated. Except for the demographic questions e.g. sex, academic semester etc., the questionnaire contained questions for the six dimensions described above (Table 2). The first four questions were open, some questions had specific answers whereas most of the questions had 5-scale answers (not at all, little, average, much, very much). In Table 2, one can see the questions as well as the type of the answers.

**Table 2:** The questions of the questionnaires categorized into six dimensions

Platform—general questions	Coursework frequency at your department (weekly)—*Open Question*
	Frequency of usage of the electronic platform Open eClass (weekly)—*Open Question*
	At which academic year did you learn to use Open eClass?—*Open Question*
Student	What internet connection do you have in your house?—*Open Question*
	How familiar are you with computers?—*5*-*scale answers*
	How familiar are you with the asynchronous e-Learning systems?—*5*-*scale answers*
Teacher	What do you think that the professor/teacher should take into account in order to make his/her teaching more effective?—*Open Question*
Design	Are you satisfied with the remote monitoring (e-Learning) of the education process through Open eClass?—*5*-*scale answers*
Courses	How many times have you visited Open eClass?—*Open Question*
	Why did you decide to attend online courses through Open eClass?—*Open Question*
	Were there any reasons that prevented you from monitoring online courses?—*Open Question*
	Did the availability and the promotion of the educational material help you?—*5*-*scale answers* Justify your answer—*Open Answer*
	How sufficient was the educational material in relation to the courses’ requirements/demands?—*5*-*scale answers*
	Where did the video lectures help you?
	Avoiding transportation to the University □
	Better revision and homework after having physically attended the course □
	Not being obligated to take notes during the physical attendance in the course and better attention to the course □
	Being informed about the days I missed physical attendance to the course □
	Did not help □
	How much did the video lectures help you for the final exams?—*5*-*scale answers*
	What do you think of the current status of the open electronic courses via Open eClass?
	Adequacy and good organization □
	Good but needs more courses □
	Good but the courses’ material have to enhanced □
	Good but the courses’ organization could be better □
	I don’t use the platform at all □
	How satisfied are you with the video lectures in relation to the requirements of the course?—*5*-*scale answers*
	Which do you think that is better: face to face teaching, e-Learning or combination of the two?—*3*-*scale answers*
Environment	Did the video lectures prevent you from attending classes at your University?—*3*-*scale answers (yes, no, maybe)*
	Do you find attractive the idea of the existence of video lectures?—*5*-*scale answers*
	Are you interested in the existence of video lectures for workshops?—*5*-*scale answers*
	Are you more interested in attending via internet video lectures or video workshops?—*3*-*scale answers (video lectures, video workshops, both)*
	Would you be interested in the creation of a chat-room (via Open eClass) to chat with the instructors?—*5*-*scale answers*
	Would you find interesting the idea of creating a forum?—*5*-*scale answers*
	Does the existence of the electronic material online help you cooperate with your classmates?—*5*-*scale answers*
Technology	What do you think about the technology? Do you believe that it sufficiently permitted the transmission of the course via Internet?—*Open Question*

There were 500 questionnaires distributed to the departments of the three islands of TEI of Ionian Islands. Generally, 150 questionnaires were distributed to the large departments of Environmental Technology (Zakynthos Island) and Business Administration (Lefkada Island) and 200 in the three departments of Cephalonia. The questionnaires were distributed to different courses covering all semesters and academic years of each department equally but without having previously reviewed the students attending each course. As a result, a course by each academic semester in each department was selected and a list of courses covering all semesters was compiled. The sample can be considered to be randomly selected and being representative covering all different ages, sex and department of study. The questionnaires were distributed during the courses and the collected questionnaires were further analyzed to evaluate Open eClass in terms of the dimensions that are related to the student, teacher, course, technology, system design, and environment. From the 500 questionnaires that were distributed, 324 were collected from all the departments of the TEI of Ionian Islands.

**Fig. 3:**
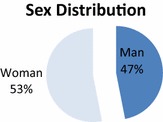
Distribution of the participants in the evaluation experiment according to their age and sex

**Fig. 4:**
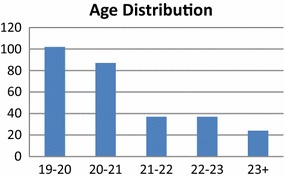
Distribution of the participants in the evaluation experiment according to their age and sex

**Fig. 5:**
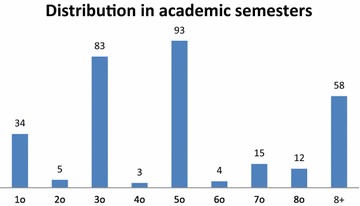
Distribution in academic semesters

From the 324 questionnaires that were collected during the evaluation experiment, 152 were answered by male students and 172 questionnaires were answered by female students. The age distribution of the students answering the questionnaires is shown in Fig. 3. Furthermore, Fig. 4 shows the distribution of the students answering the questionnaires with respect to their age. Finally, in Fig. 5 one can see the distribution of students in different semesters. More specifically, the figure shows which academic semester the students have completed. The three Figs. (3, 4, 5) prove that the sample had representatives from all academic semesters, all ages and sex. As a result the conclusions that would be drawn would be representative.

Generally, the students participating the experiment got familiar with Open eClass in the first year of their studies (88 %) and use it since then as a supplement of the traditional coursework. Attendance is optional for theoretical lessons but not for workshops. Taking into account this fact, most of students (53 %) attend classes 4–5 times a week and visit the electronic platform just 2–3 times a week (45 %). This shows that the system is used as a supplementary tool but does not have the same relevance to lectures and cannot in any case replace the role of the teacher and the face to face communication.

As far as the student dimension is concerned, the participants were asked about their familiarity with computers and the Internet. Most of the students indicated that they are familiar with computers. Most specifically, 87 % of the students stated that they are familiar with computers more than average. Additionally, 58 % of the students stated that were also quite familiar with asynchronous e-Learning systems. However, almost a quarter of the students (26 %) did not have an internet connection at home.

As far as the teacher dimension is concerned, the students were asked about the actions s/he should take to improve the course. Some of the suggestions involved improvement of his/her communication skills (27 %), personalization of the educational process by taking into account the level of its students (23 %) and simplification of the course (15 %).

Almost half of the students (55 %) stated that they were not satisfied with the e-Learning provided by the platform. This is not really surprising as the platform in 2014 has been enhanced with educational material and information about the courses. The rest of the users were averagely satisfied with the platform (37 %) and only a few were very satisfied.

The courses dimension involved several questions and revealed that 84 % of students thought that there was no preventing reason for not monitoring online courses. Additionally, the visits to the electronic platform by 79 % of the students participating in the evaluation experiment range from average to many times. The main reasons for students to visit Open eClass to view the supplementary material for the courses were further reading (32 %), a missed lecture (18 %), deficiency of the printed material (15 %) and distance (15 %). Indeed, 72 % of the students indicated that the availability and the promotion of the educational material in Open eClass helped them more than enough.

41 % of students found averagely sufficient the educational material in relation to the courses’ requirements/demands and another 23 % found it very sufficient. 58 % of the students asked during the evaluation experiment think that the development of the open electronic courses via Open eClass was good, while asking for better organization and enrichment of the educational material. 12 % thought that the material provided and the organization was sufficient. 27 % asked for more courses in Open eClass and only 3 % stated that they did not use the platform at all and read the educational material only through books and printed notes.

A main part of the questions that evaluated the courses dimension was about the video lectures. Students stated that they were not so satisfied (90 %) with video-lectures in relation to the requirements of the course, but they were highly valued as a further reading material (29 %) or note-taking supplement (21 %). These results are rather expected due to the fact that at the time the evaluation experiment took place, the departments of TEI in Cephalonia Island had no course in Open eClass with uploaded video lectures and the departments in Lefkada Island and Zakynthos Island only had one in each department. As a result, only a few users were familiar with the video lectures in the educational platform. The main conclusion drawn by the whole experiment is that most of the students (74 %) prefer a combination of the face to face teaching and e-Learning.

One of the main concerns of the teachers of TEI was that if they made video lectures of their lessons then the students would not participate in the courses actively. Contrary to what these teachers thought, 86 % of students did not think that the video lectures really prevented them from attending classes at University but used them mainly during the final exams period as an additional reading material (53 %).

67 % of students found really attractive the idea of the existence of video lectures for all courses uploaded in Open eClass and 76 % of the students also stated that it would be interesting to have video lectures for workshops. Almost half of the students completing the questionnaires would like to have both video lectures and video workshops. However, the students that prefer video lectures only for the theoretical lessons (31 %) were more than the students that prefer video lectures for the workshops (15 %).

The students also stated a preference for collaboration with each other through the platform. More specifically, 79 % of students would like to have a chat-room and 79 % of them would like to have some kind of asynchronous communication, such as a forum. Indeed, the educational material provided through the platform proved to be a mean of collaboration between the students according to the 74 % of the students participating the experiment.

With respect to the technology dimension, 74 % of the students believe that technology permitted sufficiently enough the transmission of the course via Internet.

## Conclusions

In this paper, we tried to evaluate Open eClass as a mean for providing blended learning in an institution for higher education in Greece. For this purpose, a review of the different studies, which were associated with the usage of Open eClass in Universities and TEIs in Greece, has been conducted and focuses mainly on the main advantages and disadvantages derived by its usage in all institutions. This review has revealed several studies that check a specific dimension of Open eClass but most of these studies do not provide exact data and statistical analysis. Therefore, the review reveals a shortage of integrated evaluation experiments that take into account several dimensions of LMS.

Open eClass has been established in the TEI of Ionian Islands since 2006. However, only in 2014 there has been a systematic effort to organize courses according to a specific template, review copyrights and enhance the educational material with video lectures. During the spring semester of the academic year 2014–2015, in the TEI of Ionian Islands, we conducted an evaluation experiment for Open eClass. The experiment aimed at evaluating the platform based on a six dimensions model proposed by Sun et al. (2008).

The special ‘character’ of this educational institution that has its departments spread across three different islands increases the need for an educational platform that provides learning material via the web. In this case the departments may share material and the tutors can more easily cooperate with each other and promote their courses to the students. As a result the evaluation experiment has also shown that Open eClass had been used by students to collaborate with each other as well as with their teachers.

Generally, the analysis of usage of Open eClass in institutions for Higher Education in Greece revealed the positive effects of the LMS in students and instructors (e.g. Georgouli et al. 2005; Giannelou et al. 2005; Tzitzolaki et al. 2014 etc.). An advantage that was revealed by the usage of Open eClass was the improvement of the teaching quality by promoting blended learning. Indeed, in many other studies it was reported that the platform played an important role in the educational process as a supplementary tool (e.g. Spathopoulos 2007).

According to the study that we conducted, most students get familiar with the platform in the first year of their studies. The interest of students for innovative methods is also pointed by Tsiakas et al. (2005). The majority of the students uses it as a supplementary tool but Open eClass cannot in any case replace the role of the teacher and the face to face communication with him/her. They generally use the platform to acquire information for further reading, a missed lecture etc. This conclusion conforms to the conclusions of the study implemented by Spathopoulos (2007) in TEI of Chalkida, which referred to a virtual class as a supplement to the traditional one.

Almost half of the students in our evaluation experiment stated that they were not satisfied with the distance learning provided by the platform, which is rather expected as the platform is not enhanced with rich educational material. Furthermore, this is not only the situation of TEI of Ionian Island in Greece. More specifically, Papachristos et al. (2010), in an extensive study on the use of the platform, have observed that in most cases the material and multimedia provided by such tools are poor.

Another problem of the platform identified in the literature (Papachristos et al. 2010) and confirmed by this research is the lack of availability of courses in foreign languages, which does not give any opportunity to attract international students. Furthermore, the usage of mobile application has been overlooked although the students use such devices quite often.

The students of TEI of Ionian islands were also asked about the video lectures. Video lectures were available only in a few courses and, therefore, were not considered adequate and satisfying. Indeed, the evaluation experiment revealed that most of the students prefer a combination of the face to face teaching and e-Learning. Of course, there had been studies (e.g. Vernadakis et al. 2009) that had less positive perceptions towards blended learning but this may be depending on the way blended learning is implemented.

Although, the students seem to prefer face to face communication, video-lectures were highly valued as a further reading material or note-taking supplement. One of the main concerns of the teachers of TEI was that if they made video lectures of their lessons then the students would not participate in the courses actively. Contrary to these negative perceptions, most of the students thought that the video lectures did not really prevent them from attending classes at University but helped them complementary to the face to face lecture. This conclusion conforms to the conclusions drawn by the studies of Chalkias and Anagnostopoulos (2004) and Nikolaidou et al. (2010) in Harokopio University but not agree with Verdanakis et al. (2009). The evaluation revealed that students wanted the video lectures to be expanded in workshops and not only in theoretical lessons. An interesting dimension of the platform revealed by the students is that the platform can promote collaboration by providing tools for synchronous or asynchronous communication. This collaboration was also referred in the studies of Leventidis et al. (2005) and Georgouli et al. (2005).

It is among our future plans to expand further Open eClass in TEI of Ionian Islands by implementing more courses and video lectures and run a more general evaluation experiment after two years so that students have the time to get more familiarized with the platform and the educational material. The future experiment will mainly focus on the impact of the use of the on-line platform in the academic results achieved by students.
